# Systemic inflammatory biomarkers CAR and IL-6: correlation with neurological deficit and short-term prognosis of intracerebral hemorrhage

**DOI:** 10.3389/fneur.2026.1915042

**Published:** 2026-08-24

**Authors:** Yongjian Tian, Jun Liu, Dechun Zhu, Cui Li, Yue Liu, Wei Wang

**Affiliations:** 1 Department of Laboratory Medicine, Fuyang People's Hospital, Fuyang, Anhui, China; 2 Department of Neurosurgery, Fuyang People's Hospital, Fuyang, Anhui, China

**Keywords:** C-reactive protein to albumin ratio, Glasgow Coma Scale score, interleukin-6, intracerebral hemorrhage, prognosis

## Abstract

**Background:**

Intracerebral hemorrhage (ICH) carries high mortality, with neuroinflammation driving adverse outcomes. The C-reactive protein (CRP)-to-albumin (ALB) ratio (CAR) and interleukin-6 (IL-6) are promising neuroinflammatory markers for acute brain injury, yet their combined value for evaluating ICH injury severity and 28-day mortality prediction remains unclear.

**Methods:**

We enrolled 125 ICH patients admitted to Fuyang People’s Hospital (July 2024–May 2026), split into 28-day survivors (*n* = 77) and non-survivors (*n* = 48). Admission serum CRP, ALB and IL-6 were tested. GCS score assessed brain injury severity, and ICH score stratified 28-day death risk. Baseline data were compared between groups. Spearman correlation analyzed links of CAR, IL-6 with GCS and ICH scores. Multivariate logistic regression identified independent predictors; ROC curve analysis was performed to evaluate the predictive performance of CAR, IL-6, and their combined model.

**Results:**

Serum CAR and IL-6 were markedly higher in 28-day non-survivors and rose progressively with aggravated brain injury and higher ICH mortality risk. The two biomarkers showed moderate negative correlations with GCS scores and weak positive correlations with ICH scores (all *p* < 0.05). Age, ICH score, GCS, CAR and IL-6 were significant in univariate regression. Separate multivariate logistic regression models adjusted for age, hematoma volume and admission GCS score showed that CAR (OR = 1.540, 95% CI: 1.092–2.172, *p* = 0.014) and IL-6 (OR = 3.940, 95% CI: 1.034–15.003, *p* = 0.044) independently predicted 28-day mortality. The combined CAR-IL-6 model yielded a raw AUC of 0.810, with individual AUC values of 0.702 for CAR and 0.718 for IL-6. DeLong’s test revealed it performed significantly better than CAR alone (*p* = 0.035), and the model achieved a maximum Youden index of 0.558.

**Conclusion:**

Serum CAR and IL-6 independently correlate with brain injury severity and 28-day mortality after ICH, acting as easily accessible predictive biomarkers. Prospective research is necessary to verify their clinical utility for early risk stratification.

## 1 Introduction

Intracerebral hemorrhage (ICH), a severe subtype of stroke characterized by blood extravasation into the brain parenchyma, is associated with high morbidity, mortality, and disability rates. Clinically, conventional therapeutic strategies including hematoma evacuation and blood pressure regulation are widely adopted for ICH management. However, patients frequently suffer from various complications, and the 28-day mortality rate of ICH ranges from 30 to 50% (1, 2). Early and accurate evaluation of brain injury severity and timely identification of high-risk patients prone to poor outcomes, as well as targeted stratified diagnosis and treatment, are essential for improving the prognosis of ICH patients. The infiltration of blood components into brain tissues following ICH onset triggers a cascade of inflammatory responses. This pathological process serves as the fundamental cause of parenchymal brain damage and a critical contributor to subsequent adverse clinical outcomes (3, 4). Accordingly, inflammatory biomarkers hold great potential for assessing brain injury severity and predicting clinical prognosis in ICH patients.

The C-reactive protein (CRP) to albumin (ALB) ratio (CAR) is a novel inflammatory and nutritional biomarker that comprehensively reflects the pro-inflammatory properties of CRP, as well as the anti-inflammatory function and nutritional status represented by ALB (5, 6). As a pivotal pro-inflammatory cytokine, interleukin-6 (IL-6) rises sharply in the early stage of ICH, and its expression level is significantly correlated with disease severity and hematoma expansion rate (7). The Glasgow Coma Scale (GCS) is a standardized scoring system for clinically assessing the consciousness level of patients with brain injury and has been universally applied to grade the severity of brain damage in ICH patients (8). The ICH score is routinely used to predict 30-day mortality in patients with ICH (9), yet it cannot reflect the systemic inflammatory burden following bleeding. Serum CAR and IL-6, as inflammatory biomarkers, could complement the ICH score to improve early risk stratification. Therefore, this study aimed to explore correlations of CAR and IL-6 with GCS, ICH score and 28-day mortality in ICH patients, supporting early clinical intervention and prognostic evaluation.

## 2 Materials and methods

### 2.1 General information

**Figure 1:**
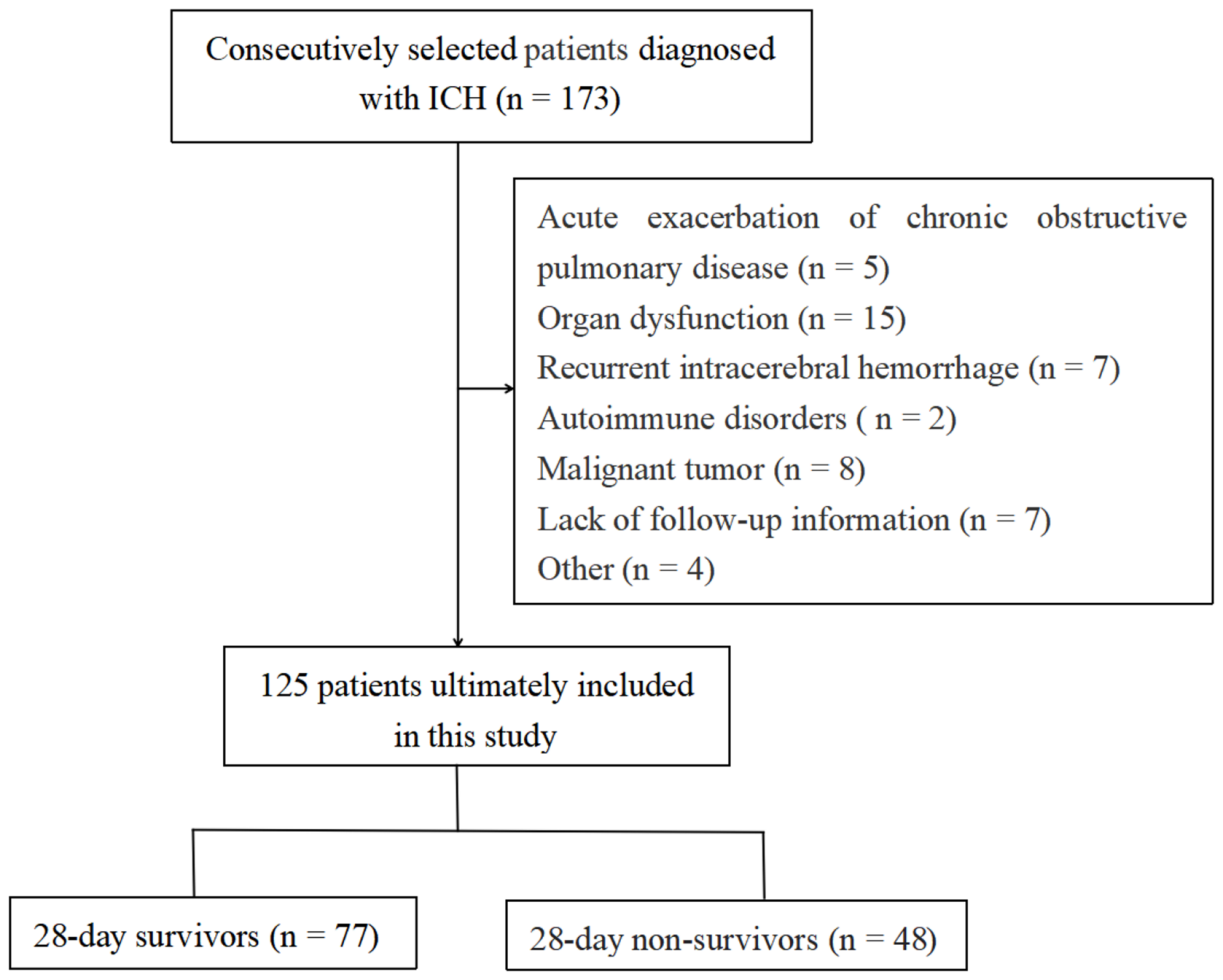
Flowchart of participants’ inclusion and exclusion criteria.

This retrospective observational study consecutively enrolled a total of 125 adult patients diagnosed with ICH admitted to Fuyang People’s Hospital between July 2024 and May 2026. The inclusion criteria were as follows: (1) diagnosis of ICH confirmed by neuroimaging examination in accordance with the established diagnostic criteria (10); (2) age ≥ 18 years old; (3) first-ever intracerebral hemorrhage. The exclusion criteria included: (1) complicated cardiac, hepatic, or renal insufficiency; (2) pre-existing infectious diseases upon admission; (3) concomitant hematological or autoimmune diseases; (4) incomplete clinical data; (5) loss to follow-up. The patient screening process is summarized in Figure 1. This study was approved by the Ethics Committee of Fuyang People’s Hospital (approval number: 2024-99) and conducted in accordance with the Declaration of Helsinki.

### 2.2 Sample size calculation

All sample size calculations were performed using PASS 15.0 software, based on the expected AUC for predicting 28-day mortality among patients with ICH. According to previous relevant studies, the expected AUC of a single inflammatory biomarker for predicting adverse outcomes after hemorrhagic stroke was approximately 0.70; we set the target AUC of combined CAR and IL-6 at 0.80. Setting *α* = 0.05 (two-sided test) and statistical power (1 − *β*) = 0.80, the ratio of non-survivors to survivors at 28 days was assumed to be 1:1.6 (consistent with the observed 48:77 ratio in this study). Using the sample size formula for receiver operating characteristic (ROC) analysis, the minimal required sample size was calculated as 106 cases. Considering potential loss to follow-up, incomplete data and exclusion of ineligible patients (estimated dropout rate 20%), the target enrollment number was set to 133 patients. Finally, a total of 173 consecutive ICH patients were initially screened, and 125 eligible subjects were included after exclusion, which met the pre-calculated sample size requirement.

### 2.3 Baseline data collection

Baseline demographic and clinical data of all participants were collected from electronic medical records, including gender, age, smoking and drinking history, and underlying medical histories such as hypertension, diabetes and prior stroke. We also recorded key ICH-related variables: admission blood pressure, onset-to-admission interval, raw admission GCS score, ICH score, hematoma volume, hemorrhage location, intraventricular extension, treatment modality, and the occurrence of in-hospital pulmonary infection developed after admission.

### 2.4 Laboratory index detection

All venous blood samples for CRP, ALB and IL-6 testing were collected within 24 h of emergency admission, before any definitive interventions were administered, including hematoma evacuation, burr hole drainage, decompressive craniectomy, mannitol dehydration, and intravenous anti-inflammatory supportive therapy. Blood samples were kept at room temperature for 30 min to allow clot formation, the blood samples were centrifuged at 3,000 r/min for 10 min to separate the serum. Serum CRP and IL-6 levels were measured using the Jet-iStar3000 immunoassay analyzer (Joinstar Biomedical Technology Co., Ltd., Hangzhou, China). The linear range of IL-6 assay was 3.0–5000.0 pg./mL with *r* ≥ 0.990, and the total CV ≤ 10.0%. Serum ALB was quantified on the VITROS 5600 Integrated System (Ortho Clinical Diagnostics, USA). All detection reagents and consumables were original supporting products, and all experimental operations were strictly performed in accordance with the manufacturer’s instructions. The CAR was calculated as CRP level divided by ALB level.

### 2.5 GCS score evaluation

The GCS score was applied to assess the neurological consciousness level of ICH patients (11). The scale consists of three items: eye opening response, verbal response, and motor response, with a total score ranging from 3 to 15 points. Brain injury severity was classified as mild (13–15 points), moderate (9–12 points), and severe (3–8 points).

### 2.6 ICH score evaluation

Upon admission, the original ICH score of each patient was calculated based on cranial CT findings and clinical data. The scoring system contained five items: GCS, age, hematoma volume, hemorrhage location, and intraventricular hemorrhage. The total score ranged from 0 to 6, with higher scores indicating higher risk of short-term death. GCS: 13–15 points = 0; 5–12 points = 1; 3–4 points = 2; Age: < 80 years = 0; ≥ 80 years = 1; Hematoma volume: <30 mL = 0; ≥30 mL = 1; Bleeding site: supratentorial hemorrhage = 0; infratentorial hemorrhage = 1; Intraventricular hemorrhage: absent = 0; present = 1 (9).

### 2.7 Clinical outcomes

All enrolled patients were followed up from admission until death or the 28th day after ICH onset (whichever occurred earlier). The 28-day survival status was verified through inpatient electronic medical records for hospitalized patients and telephone interviews for discharged patients. The exact time of death was recorded for non-survivors. Patients who were lost to follow-up or withdrew from the study midway were excluded, and all enrolled subjects had complete follow-up data.

### 2.8 Statistical analysis

SPSS 27.0 statistical software was used for data processing and statistical analysis. Quantitative data conforming to normal distribution were presented as mean ± standard deviation (SD), and independent-sample *t*-test was adopted for comparison between two groups. Quantitative data with skewed distribution were expressed as interquartile ranges (IQR), and Mann–Whitney *U* test was used for two-group comparison, while Kruskal–Wallis *H* test was applied for multi-group comparison. Categorical data were described as case number and percentage [*n* (%)], and chi-square (*χ*^2^) test was performed for inter-group comparison. Spearman rank correlation analysis was performed to evaluate correlations of serum CAR and IL-6 levels with admission GCS and ICH scores indicating brain injury severity. A multivariate binary logistic regression model was constructed to identify independent predictors of 28-day mortality. CAR was incorporated as a continuous variable using raw measured values without transformation. IL-6 was log_10_-transformed and analyzed as a continuous variable. Variables with *p* < 0.20 in univariate analysis were entered into multivariate regression, whereas variables demonstrating severe collinearity were excluded. Subsequently, a combined CAR-IL-6 logit model was established via binary logistic regression using raw CAR and log_10_-transformed IL-6 as predictors. The formula was: Combined model = −3.328 + 0.327 × CAR + 1.214 × log_10_(IL-6). ROC curves were plotted to assess predictive performance of CAR, IL-6 and their combined model. Stratified 10-fold cross-validation was used for internal validation, and the DeLong’s test was applied to compare raw AUC values from the original dataset. A two-tailed *p* < 0.05 was defined as statistically significant.

## 3 Results

### 3.1 General clinical characteristics

Baseline clinical data were compared between 77 survivors and 48 non-survivors at 28 days. Non-survivors were significantly older, presented with lower admission GCS scores and higher ICH scores (all *p* < 0.05). Graded brain injury severity and ICH score-based risk strata also differed markedly between the two groups (both *p* < 0.001). No other baseline variables exhibited statistically significant intergroup differences (all *p* > 0.05). Full details are provided in Table 1.

**Table 1:** Comparison of general clinical data and research indicators between the two groups.

Variable	28-day survivors (*n* = 77)	28-day non-survivors (*n* = 48)	*p-*value
Age, years, median (IQR)	58.00 (50.75,69.25)	64.00 (55.00,76.50)	0.023
BMI, kg/m^2^, mean ± SD	23.41 ± 1.76	22.89 ± 2.20	0.142
Sex, *n* (%)			0.718
Male	49 (63.64%)	29 (60.42%)	
Female	28 (36.36%)	19 (39.58%)	
Tobacco smoking, *n* (%)	35 (45.45%)	21 (43.75%)	0.852
Diabetes mellitus, *n* (%)	18 (23.38%)	8 (16.67%)	0.369
Hypertension, *n* (%)	70 (90.91%)	44 (91.67%)	0.884
Coronary atherosclerotic heart disease, *n* (%)	18 (23.38%)	8 (16.67%)	0.471
Drink alcohol, *n* (%)	24 (31.17%)	18 (37.50%)	0.466
Hematoma volume, mL, mean ± SD	44.25 ± 12.73	48.85 ± 16.99	0.087
Hematoma location, *n* (%)			0.183
Basal ganglia	42 (54.55%)	21 (43.75%)	
Lobar	12 (15.58%)	10 (20.83%)	
Thalamus	12 (15.58%)	3 (6.25%)	
Cerebellum	5 (6.49%)	5 (10.42%)	
Brainstem	2 (2.60%)	5 (10.42%)	
Multifocal hematoma	4 (5.20%)	4 (8.33%)	
Intraventricular hemorrhage, *n* (%)	30 (38.96%)	23 (47.92%)	0.324
Admission systolic blood pressure, mmHg, mean ± SD	170.65 ± 28.93	171.37 ± 31.12	0.895
Admission diastolic blood pressure, mmHg, mean ± SD	96.01 ± 19.15	95.97 ± 21.77	0.993
Onset-to-admission time, hour, median (IQR)	2.(2.0,3.5)	3 (2.0,4.0)	0.343
Admission GCS score, mean ± SD	11.45 ± 2.85	8.17 ± 3.77	<0.001
Pulmonary infection during hospitalization, *n* (%)	63 (81.82%)	36 (75.00%)	0.361
Treatment modality, *n* (%)			0.115
Conservative management	12 (15.59%)	11 (22.92%)	
Hematoma evacuation	36 (46.75%)	12 (25.00%)	
Burr hole drainage	14 (18.18%)	12 (25.00%)	
Hematoma evacuation combined with decompressive craniectomy	15 (19.48%)	13 (27.08%)	
Brain injury severity (stratified by admission GCS score), *n* (%)			<0.001
Mild brain injury (GCS 13–15)	41 (53.25%)	12 (25.00%)	
Moderate brain injury (GCS 9–12)	28 (36.36%)	20 (41.67%)	
Severe brain injury (GCS 3–8)	8 (10.39%)	16 (33.33%)	
Admission ICH score, mean ± SD	1.43 ± 0.82	3.19 ± 0.96	<0.001
ICH mortality risk grade (based on admission ICH score), *n* (%)			<0.001
Low risk (0–1)	39 (50.65%)	1 (2.08%)	
Moderate risk (2)	32 (41.56%)	9 (18.75%)	
High risk (≥3)	6 (7.79%)	38 (79.17%)	

GCS, Glasgow Coma Scale; BMI, body mass index; ICH, intracerebral hemorrhage.

### 3.2 Comparison of serum CRP, ALB, CAR and IL-6 levels between 28-day survivors and non-survivors

**Figure 2:**
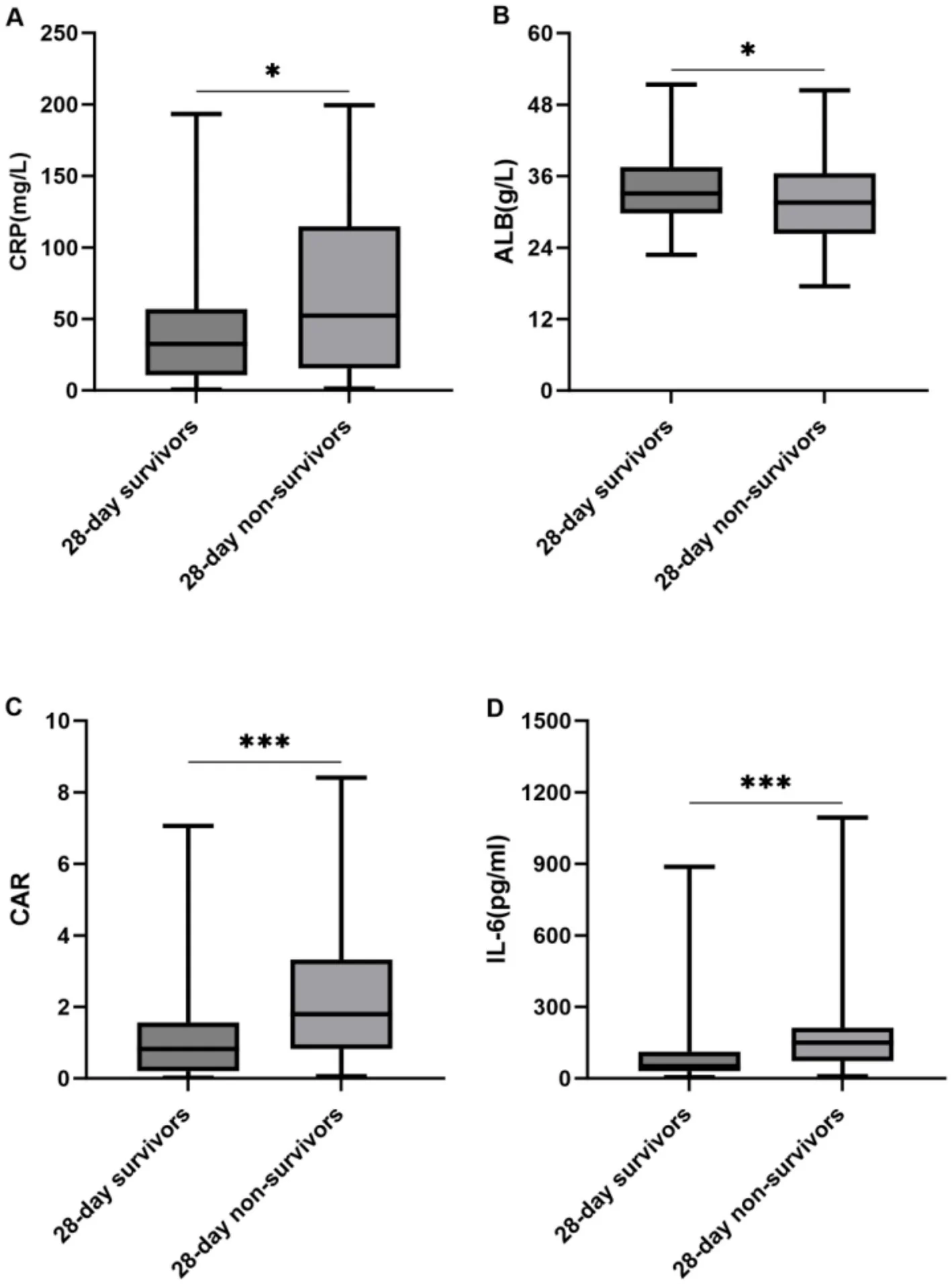
Box plots comparing serum CRP, ALB, CAR, and IL-6 levels between 28-day survivors and non-survivors with ICH **(A)** CRP; **(B)** ALB; **(C)** CAR; **(D)** IL-6. *p < 0.05, ***p < 0.001.

Inflammatory and nutritional biomarker levels were compared between 28-day survivors and 28-day non-survivors using box plots (Figure 2). Serum CRP levels were significantly higher in 28-day non-survivors than in 28-day survivors (*p* < 0.05; Figure 2A). Conversely, serum ALB levels were significantly lower in 28-day non-survivors (*p* < 0.05; Figure 2B). Both CAR and IL-6 levels were markedly elevated among 28-day non-survivors, with highly significant intergroup differences (both *p* < 0.001; Figures 2C,D).

### 3.3 Distributions of serum CAR and IL-6 levels stratified by GCS and ICH scores

As shown in Table 2, serum CAR and IL-6 increased stepwise with elevated brain injury severity (stratified by GCS scores) and higher 28-day mortality risk (stratified by ICH scores). Differences in CAR and IL-6 among GCS subgroups were all *p* < 0.001; for ICH risk strata, *p* = 0.018 for CAR and *p* < 0.001 for IL-6.

**Table 2:** Serum CAR and IL-6 levels were stratified separately by admission GCS scores (for brain injury severity subgroups) and ICH scores (for mortality risk strata).

Variable	CAR	IL-6 (pg/mL)
Brain injury severity (stratified by admission GCS scores)		
Mild brain injury (*n* = 53)	0.62 (0.17,1.37)	32.40 (19.05,51.50)
Moderate brain injury (*n* = 48)	1.29 (0.45,2.27)	134.15 (78.10,185.35)
Severe brain injury (*n* = 24)	2.22 (1.21,4.76)	197.81 (127.55,532.60)
*p-*value	<0.001	<0.001
ICH risk stratification (based on admission ICH scores)		
Low risk (*n* = 40)	0.86 (0.13,1.41)	46.33 (20.88,111.53)
Moderate risk (*n* = 41)	1.12 (0.39,2.58)	54.45 (32.70,120.50)
High risk (*n* = 44)	1.66 (0.62,2.72)	144.84 (72.55,195.15)
*p-*value	0.018	<0.001

CAR, C-reactive protein to albumin ratio; IL-6, interleukin-6; GCS, Glasgow Coma Scale; ICH, intracerebral hemorrhage.

### 3.4 Spearman correlations of serum CAR and IL-6 with raw admission GCS and ICH score

Spearman correlation analysis revealed that CAR and IL-6 were moderately negatively correlated with admission GCS scores (*r_s_* = −0.407, *p* < 0.001; *r_s_* = −0.665, *p* < 0.001). Higher biomarker levels corresponded to more severe brain injury. Both markers showed weak positive correlations with ICH scores (CAR: *r_s_* = 0.250, *p* = 0.004; IL-6: *r_s_* = 0.248, *p* = 0.005), indicating elevated CAR and IL-6 were linked to greater 28-day mortality risk (Table 3).

**Table 3:** Spearman correlations of serum CAR and IL-6 with raw admission GCS and ICH scores.

Variable	Brain injury severity (raw admission GCS scores)		ICH risk strata (raw admission ICH scores)	
	*r_s_*	*p-*value	*r_s_*	*p-*value
CAR	−0.407	<0.001	0.250	0.004
IL-6	−0.665	<0.001	0.248	0.005

CAR, C-reactive protein to albumin ratio; IL-6, interleukin-6; GCS, Glasgow Coma Scale; ICH, intracerebral hemorrhage. *r_s_* = Spearmanʼs rank correlation coefficient. Raw continuous GCS (3–15) and ICH (0–6) scores were analyzed without categorical grouping. Lower GCS = more severe brain injury; Higher ICH score = higher risk of short-term mortality. Negative *r_s_* for GCS and positive weak *r_s_* for ICH reflected the corresponding inflammatory trends.

### 3.5 Univariate and multivariate logistic regression analyses of predictors for 28-day mortality

Univariate logistic regression (Table 4) revealed that age, admission ICH scores, GCS scores, CAR and IL-6 were significantly correlated with 28-day mortality (all *p* < 0.05), while hematoma volume showed no statistical association (*p* = 0.091). Variables with *p* < 0.20 were enrolled into multivariate models. We excluded the ICH score from multivariate regression to avoid collinearity and overlapping information with age, hematoma volume and admission GCS score. Each independent model was adjusted for three predefined confounders: age, hematoma volume and admission GCS score. These two markers were fitted in separate multivariate models to avoid mutual collinearity (Table 5). After adjustment for age, hematoma volume and admission GCS score, CAR (OR = 1.540, 95% CI: 1.092–2.172, *p* = 0.014) and IL-6 (OR = 3.940, 95% CI: 1.034–15.003, *p* = 0.044) emerged as independent predictors of 28-day mortality, while hematoma volume lost independent predictive value. Age and admission GCS score remained independent prognostic factors for 28-day mortality across both models.

**Table 4:** Univariate logistic regression analysis for predictors of 28-day mortality in patients with ICH.

Variable	OR	95% CI	*p-*value
Age	1.043	1.012–1.076	0.007
Admission ICH scores	11.925	4.993–28.480	<0.001
Admission GCS scores	0.751	0.663–0.850	<0.001
Hematoma volume	1.023	0.996–1.050	0.091
CAR	1.815	1.336–2.466	<0.001
IL-6	5.673	2.315–13.903	<0.001

OR, odds ratio; CI, confidence interval; CAR, C-reactive protein to albumin ratio; IL-6, interleukin-6; ICH, intracerebral hemorrhage; GCS, Glasgow Coma Scale. All listed variables met the screening threshold of (*p* < 0.20). CAR was treated as an untransformed continuous variable, whereas log_10_ transformation was applied to raw IL-6 concentrations prior to statistical analyses.

**Table 5:** Multivariate logistic regression analysis of predictors for 28-day mortality after ICH.

Variable	Model 1 (with CAR)			Model 2 (with IL–6)		
	OR	95% CI	*p-*value	OR	95% CI	*p-*value
Age	1.048	1.010–1.087	0.012	1.064	1.006–1.124	0.029
Admission GCS score	0.787	0.686–0.903	<0.001	0.737	0.647–0.840	<0.001
Hematoma volume	1.022	0.990–1.056	0.183	1.017	0.976–1.060	0.424
CAR	1.540	1.092–2.172	0.014	—	—	—
IL-6	—	—	—	3.940	1.034–15.003	0.044

OR, odds ratio; CI, confidence interval; VIF, variance inflation factor; CAR, C-reactive protein to albumin ratio; IL-6, interleukin-6. CRP and albumin were excluded due to collinearity with CAR. Two separate multivariate logistic regression models adjusted for age, hematoma volume and admission GCS score were built for untransformed CAR and log_10_-transformed IL-6, respectively. OR for log_10_(IL-6) represent a 1-unit increment. The ICH score was excluded to avoid collinearity and overlapping covariates with admission GCS score. Each model met EPV ≥ 10, with all covariates VIF < 5.

### 3.6 Comparison of the predictive performance of CAR, IL-6 and their combined model for 28-day mortality in patients with ICH

**Figure 3:**
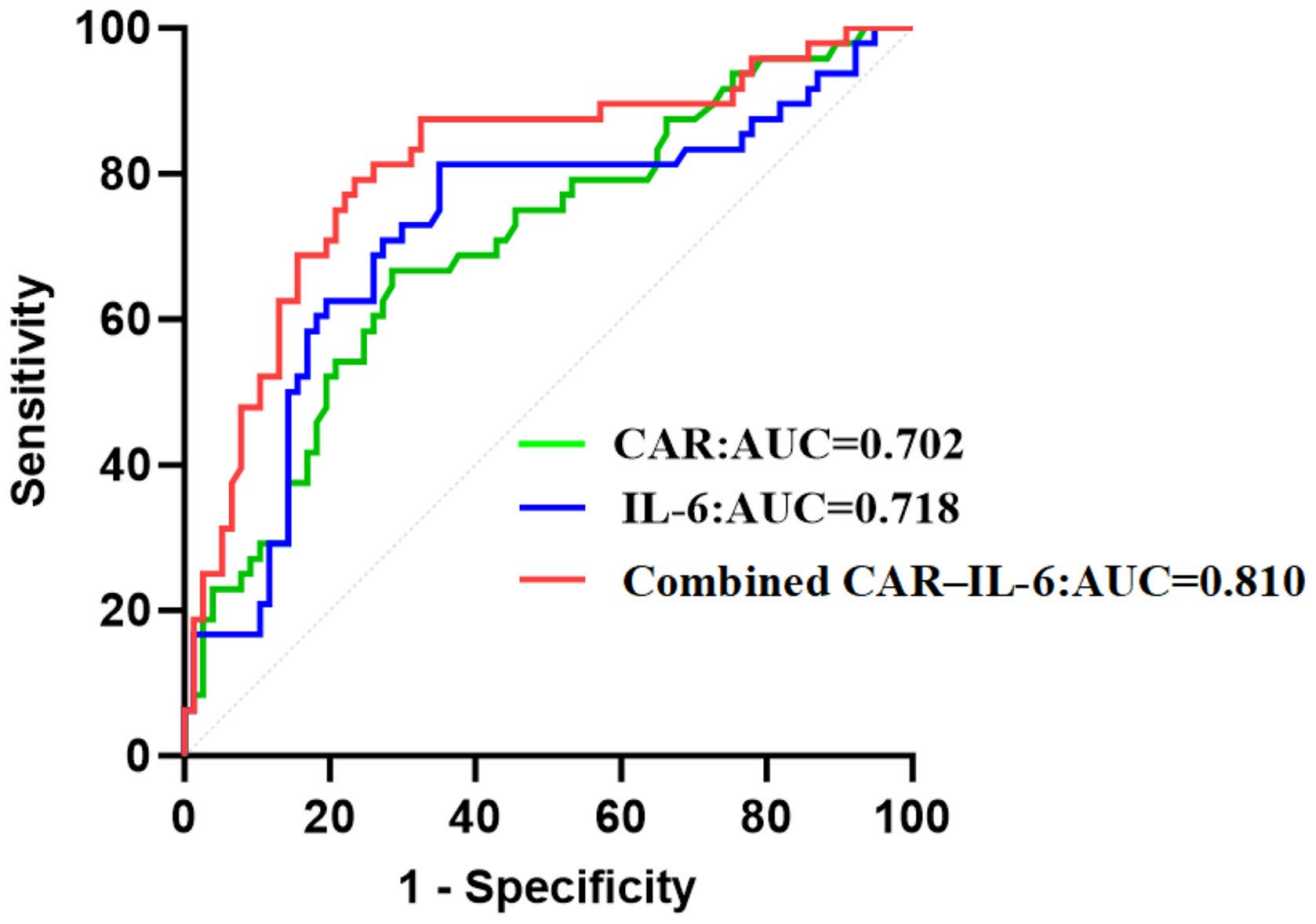
ROC curves of CAR, IL-6 and their combined model for predicting 28-day mortality in patients with ICH. The AUC values presented in the figure are raw AUC values derived from the full dataset; cross-validated AUC values obtained via stratified 10-fold cross-validation are listed in Table 7, and raw AUC values were used for pairwise comparisons using DeLong’s test. All ROC analyses for IL-6 were performed using log_10_-transformed values.

ROC analysis and corresponding diagnostic metrics were calculated to evaluate the predictive ability of CAR, IL-6 and their combined model for 28-day mortality (Figure 3 and Tables 6, 7). Stratified 10-fold cross-validation was implemented for internal validation. Pairwise comparisons using the DeLong’s test were conducted exclusively on raw AUC values from the full dataset. Raw AUC values were 0.810 for the combined model, 0.702 for CAR, and 0.718 for IL-6 (Figure 3). The cross-validated AUC values were 0.798 for the combined CAR-IL-6 model, 0.700 for CAR and 0.736 for IL-6. The modest discrepancy between raw and cross-validated AUC values indicated no obvious model overfitting. The combined model exhibited significantly better predictive performance than CAR alone (*p* = 0.035), with a borderline non-significant difference versus IL-6 (*p* = 0.053). As shown in Table 6, the combined model yielded the maximum Youden index (0.558), with a sensitivity of 79.17% and specificity of 76.62%.

**Table 6:** Diagnostic performance of CAR, IL-6 and their combined model for predicting 28-day mortality.

Variable	Sensitivity	Specificity	Cut-off value	Youden index
CAR	66.71%	71.43%	1.370	0.381
IL-6	81.25%	64.94%	1.797	0.462
Combined CAR-IL-6	79.17%	76.62%	—	0.558

CAR, C-reactive protein to albumin ratio; IL-6, interleukin-6; AUC, area under the curve. The optimal cut-off value of 1.797 for IL-6 corresponds to log_10_-transformed data, with the original serum concentration of 62.70 pg/mL.

**Table 7:** AUC values of CAR, IL-6 and their combined model for predicting 28-day mortality.

Variable	Raw AUC	Cross-validated AUC	95% CI	*p-*value (*vs* combined score, DeLong’s test)
CAR	0.702	0.700	0.614–0.780	0.035
IL-6	0.718	0.736	0.631–0.795	0.053
Combined CAR-IL-6	0.810	0.798	0.730–0.874	—

CAR, C-reactive protein to albumin ratio; IL-6, interleukin-6; AUC, area under the curve. Raw AUC: ROC AUC calculated on the full dataset; Cross-validated AUC: mean AUC from stratified 10-fold cross-validation for overfitting assessment. 95% CI: 95% confidence interval of raw AUC. DeLong’s test was applied only to compare raw AUC values with those of the combined CAR-IL-6 model. “—” denotes no comparison for the combined model itself.

## 4 Discussion

ICH is a life-threatening acute cerebrovascular disease with high mortality. Systemic inflammatory response is a crucial mechanism that exacerbates disease progression; thus, effective inflammatory markers are urgently needed for disease assessment, prognostic prediction, and reducing 28-day mortality (11). CAR can comprehensively reflect systemic inflammation and nutritional status, while IL-6, as a key pro-inflammatory factor, mediates early secondary brain injury. Combining these two indicators to evaluate 28-day mortality risk in ICH patients has practical value for clinical diagnosis, treatment, and prognostic prediction.

Follow-up data showed that the 28-day mortality rate was 38.40% (48/125) among the 125 enrolled ICH patients, which is consistent with previous studies (12). As reported by Chen et al. (13), blood extravasation after ICH initiates neuroinflammatory responses: infiltrated blood components activate systemic immunity, recruit leukocytes around the hematoma, and trigger robust release of IL-6, which drives secondary brain damage and unfavorable short-term outcomes. Consistent with Peng et al. (14), our data may suggest that 28-day non-survivors had a markedly larger proportion of moderate-to-severe brain injury and were more likely to fall into the high ICH risk group compared with survivors. Meanwhile, non-survivors presented significantly higher serum CRP, CAR and IL-6 concentrations alongside lower albumin levels (all *p* < 0.05).

Extravasated blood activates microglia and astrocytes to secrete IL-6, which further activates the NF-κB pathway and induces massive pro-inflammatory mediators (15). This inflammatory cascade impairs blood–brain barrier integrity, aggravates cerebral edema, promotes hematoma expansion and neuronal apoptosis, and consequently increases the risk of 28-day mortality. In addition, IL-6-mediated systemic inflammation stimulates hepatic CRP synthesis and decreases circulating albumin levels, which together lead to elevated CAR. We performed stratified analyses according to admission GCS and ICH scores. The results demonstrated that serum CAR and IL-6 levels rose gradually as brain injury became more severe and 28-day mortality risk increased. Spearman’s rank correlation analysis further confirmed that both biomarkers exhibited moderate negative correlations with continuous GCS scores and weak positive correlations with ICH scores. Ma et al. (16) reported that blocking the IL-6 receptor after ICH could lower systemic IL-6 concentrations. This intervention suppresses peripheral immune cell infiltration and neutrophil extracellular trap generation. Accordingly, neuroinflammation is alleviated, blood–brain barrier function is maintained, neuronal apoptosis is reduced, white matter microstructure is preserved, and long-term neurological recovery is improved after hemorrhage.

Multiple cohorts have validated CAR as a robust prognostic biomarker across cerebral infarction, sepsis and acute coronary syndrome (17–19). Elevated CAR also correlates with unfavorable outcomes after subarachnoid hemorrhage (20), while high admission IL-6 reliably reflects severe early neurological damage and higher mortality in ICH populations (21). After adjusting for age, hematoma volume and admission GCS score, multivariate binary logistic regression confirmed that elevated CAR (OR = 1.540, *p* = 0.014) and IL-6 (OR = 3.940, *p* = 0.044) independently predicted 28-day mortality. This finding echoes the report by Haferkorn et al. (22), who identified admission CAR > 1.22 as an independent predictor of in-hospital death among ICH patients; our study further identified an optimal CAR cut-off of 1.37 for predicting 28-day mortality. The discrepancy in optimal CAR cut-offs (1.22 *vs.* 1.37) may stem from differences in study endpoints: Haferkorn’s research focused on in-hospital mortality, whereas our analysis targeted 28-day all-cause mortality. Variations in patient baseline characteristics and clinical management protocols across cohorts may also contribute to this numerical difference. We further evaluated predictive performance via ROC analysis. The combined CAR-IL-6 model yielded a raw AUC of 0.810 and a cross-validated AUC of 0.798; the modest discrepancy indicated minimal overfitting, while the single-marker models yielded raw AUC values of 0.702 (CAR) and 0.718 (IL-6). Raw AUC comparisons via DeLong’s test revealed significant superiority of the combined model over CAR (*p* = 0.035). This finding suggests that integrating CAR and IL-6 can improve prognostic performance compared with single CAR measurement. Consistent with prior cerebrovascular studies that combined inflammatory ratios and interleukins for risk stratification (23), our findings further expand current evidence: CAR and IL-6 exert complementary predictive effects, jointly improving prognostic performance for 28-day mortality after ICH.

This study has several inherent limitations. First, this retrospective single-center design may introduce selection bias and restrict the generalizability of our conclusions due to the limited sample size. Second, the subgroup with severe brain injury only contained 24 participants. The small sample size of this subgroup may reduce the stability of correlation and regression results. Such a modest subgroup sample may destabilize correlation and regression statistical estimates. Third, unmeasured confounders such as pre-admission drugs, surgical plans and comorbidities could interfere with our results. Fourth, only single admission-point detection of CAR and IL-6 was performed without repeated dynamic testing. Fifth, pulmonary infection is a common ICH complication, yet its incidence showed no intergroup difference here, possibly due to standardized anti-infection protocols in our center. Further large multicenter prospective trials with long-term follow-up are needed to verify our conclusions.

## 5 Conclusion

Our study verified that serum CAR and IL-6 positively correlated with ICH severity (lower GCS scores, higher ICH scores) and independently predicted 28-day mortality, with their combination providing better prognostic performance. As a retrospective correlational analysis, this work fails to establish causality; elevated CAR and IL-6 are merely markers of post-hemorrhage inflammation rather than direct contributors to death. Prospective and interventional studies are needed to confirm our findings and elucidate causal mechanisms.

## Data Availability

The original contributions presented in the study are included in the article/supplementary material, further inquiries can be directed to the corresponding author.
